# A Rare Olive Compound Oleacein Improves Lipid and Glucose Metabolism, and Inflammatory Functions: A Comprehensive Whole-Genome Transcriptomics Analysis in Adipocytes Differentiated from Healthy and Diabetic Adipose Stem Cells

**DOI:** 10.3390/ijms241310419

**Published:** 2023-06-21

**Authors:** Rui Wang, Munkhzul Ganbold, Farhana Ferdousi, Kenichi Tominaga, Hiroko Isoda

**Affiliations:** 1 Tsukuba Life Science Innovation Program (T-LSI), University of Tsukuba, Tsukuba 305-8577, Japan; 2 Open Innovation Laboratory for Food and Medicinal Resource Engineering (FoodMed-OIL), National Institute of Advanced Industrial Science and Technology (AIST), Tsukuba 305-8577, Japan; 3 Alliance for Research on the Mediterranean and North Africa (ARENA), University of Tsukuba, Tsukuba 305-8577, Japan; 4 Faculty of Life and Environmental Sciences, University of Tsukuba, Tsukuba 305-8572, Japan

**Keywords:** oleacein, olive compound, human adipose-derived stem cells, DNA microarray analysis, lipid metabolism, anti-inflammatory effect, glucose and lipid homeostasis

## Abstract

Oleacein (OLE), a rare natural compound found in unfiltered extra virgin olive oil, has been shown to have anti-inflammatory and anti-obesity properties. However, little is known regarding the mechanisms by which OLE influences metabolic processes linked to disease targets, particularly in the context of lipid metabolism. In the present study, we conducted whole-genome DNA microarray analyses in adipocytes differentiated from human adipose-derived stem cells (hASCs) and diabetic hASCs (d-hASCs) to examine the effects of OLE on modulating metabolic pathways. We found that OLE significantly inhibited lipid formation in adipocytes differentiated from both sources. In addition, microarray analysis demonstrated that OLE treatment could significantly downregulate lipid-metabolism-related genes and modulate glucose metabolism in both adipocyte groups. Transcription factor enrichment and protein–protein interaction (PPI) analyses identified potential regulatory gene targets. We also found that OLE treatment enhanced the anti-inflammatory properties in adipocytes. Our study findings suggest that OLE exhibits potential benefits in improving lipid and glucose metabolism, thus holding promise for its application in the management of metabolic disorders.

## 1. Introduction

Adipocytes, also known as lipocytes and fat cells, originate from mesenchymal stem cells and serve the purpose of storing energy in the form of fat [1]. These cells play a crucial role in responding to the overall energy state of an organism by reacting to anabolic hormones such as insulin and catabolic hormones such as glucagon, which in turn affects the processes of lipogenesis and lipolysis [2]. Apart from its role in the systemic energy state, adipocytes also release multiple adipokines that possess crucial regulatory functions in various physiological processes, such as feeding behavior, energy expenditure, and glucose homeostasis, among others [3,4]. The maintenance of metabolic activity in healthy adipocytes is dependent on processes such as angiogenesis and mitochondrial biogenesis, as described in previous literature [5,6]. Conversely, adipocyte dysfunction can occur as a result of chronic hypoxia, inflammation, and mitochondrial dysfunction, leading to a range of conditions, including insulin resistance, hypertension, and atherosclerosis [7,8]. This imbalance is thought to be a major contributor to the development of type 2 diabetes [9].

Human adipose-derived stem cells (hASCs), which were derived from adult white adipose tissue, exhibit a fibroblast-like morphology. Under appropriate differentiation conditions, hASCs possess a high capacity to proliferate and have the potential to differentiate into various mesodermal cell lineages, such as adipocytes, chondrocytes, osteocytes, and myocytes [10]. Multiple investigations have employed hASCs as a resource for inducing adipocyte differentiation, facilitating the exploration of the impact of diverse compounds on adipocytes. For instance, saffron, crocin, and crocetin have demonstrated efficacious inhibition of adipocyte differentiation in hASCs, offering potential advantages for the prevention and management of obesity [11]. Additionally, the use of hASCs has enabled the discovery of Thymoquinone’s ability to hinder adipocyte differentiation by suppressing PPARγ and FAS protein expression, positioning it as a promising anti-obesity compound [12].

Apart from adipocytes from hASCs, diabetic human adipose tissue-derived mesenchymal stem cells (d-hASCs) were utilized as a tool to induce adipocyte differentiation. One study found that diabetic cells fail to differentiate into adipocytes first, while insulin receptor gene expression was downregulated in dASCs [13]. An additional research report suggested that the natural compounds, squalene, and its amphiphilic derivative can enhance the metabolism of adipocytes that have been differentiated from d-hASCs, thereby preventing excessive lipogenesis [14]. It can be observed that although adipocytes eventually become the same cell type of adipocytes, different sources can display distinct gene profiles during adipogenesis under various conditions.

Natural compounds have emerged as a promising therapeutic approach for obesity-related diseases, and their combination with conventional drugs has gained considerable attention. The available evidence increasingly suggests that extra virgin olive oil (EVOO) possesses notable health benefits. These include the modulation of inflammatory and immune responses, the prevention of cancer, as well as the reduction in the risk of coronary heart disease and metabolic disorders [15]. The beneficial effects of EVOO have been attributed to a wide range of phenolic compounds, including phenolic alcohols, hydroxytyrosol, tyrosol, and their secoiridoid derivatives, such as oleuropein aglycone, ligstroside aglycone, oleocanthal, and oleuropein [16]. These bioactive constituents, along with their hydrolysis derivatives, have demonstrated effectiveness in the treatment of diabetes, cardiovascular and neurological diseases, cancer, and microbial infections [17]. One in vitro study has demonstrated that oleuropein, the primary phenolic compound found in olive leaves, exhibits the capability to inhibit differentiation through the suppression of mitotic clonal expansion and downregulation of genes associated with adipogenesis [18]. There is considerable interest in investigating whether other compounds present in olives exhibit therapeutic effects, such as oleuropein, on lipid accumulation.

Oleacein (OLE) is a secoiridoid present in both the fruit and leaves of the Olea europaea L. plant (Oleaceae), as well as in EVOO. OLE is synthesized by combining the dialdehydes form of decarboxymethyl elenolic acid with hydroxytyrosol, also known as 3,4-DHPEA-EDA [19] and also can be synthesized from oleuropein [20]. For OLE functions, studies have shown that OLE possesses potent anti-inflammatory properties. OLE was found to decrease the levels of anti-inflammatory cytokines such as IL-10 in both THP-1-derived macrophages and experimental autoimmune encephalomyelitis mice [21,22]. Moreover, several studies have demonstrated the anti-obesity properties of OLE in vitro and in vivo. G. E. Lombardo et al. showed that OLE enhanced insulin sensitivity in mice fed a normal diet and reduced body weight and total serum cholesterol in diabetic mice [23]. In 3T3-L1 cells, OLE strongly inhibited lipid accumulation and lowered peroxisome proliferator-activated receptor gamma (PPARγ) and fatty acid synthase (FAS) protein levels while elevating adiponectin levels [24]. OLE reduces lipid accumulation in macrophages by activating the SRB1 receptor and the ABC transporters [25]. However, while OLE restored the expression of the insulin-sensitive muscle/fat glucose transporter Glut-4, it did not affect plasma glucose or serum triglyceride levels [23].

Currently, the effect of OLE on lipid and glucose metabolism and its potential therapeutic applications in adipocytes remains unknown, and there are insufficient data available on the underlying regulatory mechanism. Hence, our study aims to evaluate the potential effects of OLE on lipid accumulation in adipocytes. We investigated the biological effects of OLE by conducting whole-genome DNA microarray analysis on adipocytes differentiated from healthy and diabetic hASCs. Furthermore, we explored the potential preventive impact of OLE on adipocytes differentiated from diabetic donors’ adipose stem cells.

## 2. Results

### 2.1. Effect of OLE on hASCs Viability and Proliferation

First, we examined if hASCs retain their capacity to proliferate in the presence of OLE. hASCs were incubated with 0, 1, 5, 10, 20, 40, 80, and 100 μM of OLE, and an MTT assay was performed after 24 h. The viability of the treated cells relative to the vehicle control was assessed as a percentage of optical density readings. We found that OLE did not show any cytotoxicity [26]. There was no significant difference in OLE-treated hASCs compared to nontreated control cells at concentrations ranging from 0 to 40 µM, with only minimal changes in % activity. However, OLE was found to significantly stimulate cell proliferation at the concentration of 80 μM and more (Supplementary Figure S1).

### 2.2. Effect of OLE on Lipid Accumulation during Adipocytic Differentiation of hASCs

**Figure 1:**
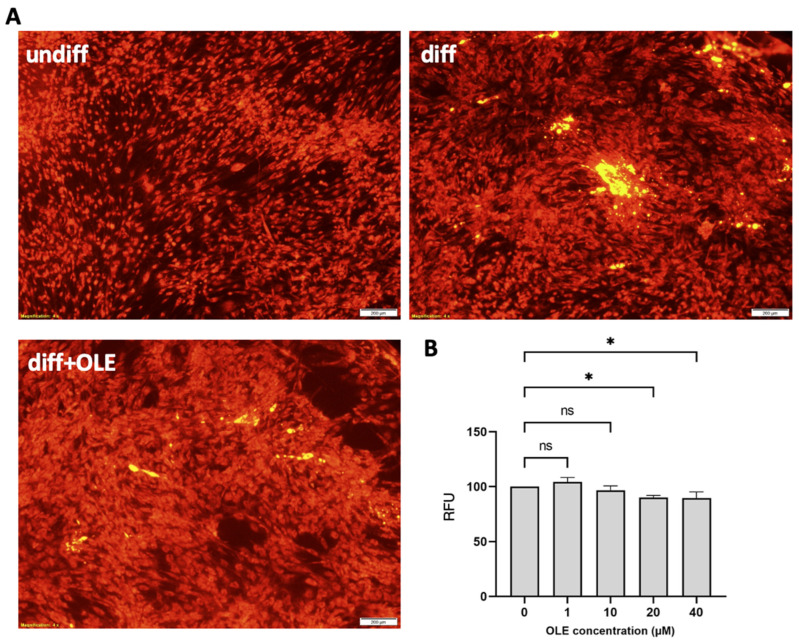
Effect of OLE treatment on cellular lipid accumulation in hASCs induced to adipocytic differentiation. Cells were cultured in an adipocytic differentiation medium for 7 days and then incubated with 20 μM of OLE for 14 days. Intracellular lipid levels were evaluated quantitatively and qualitatively using Nile red staining. (**A**) Representative fluorescence image. The scale bar indicates 200 μm. (**B**) Nile red fluorescence intensity quantification. Lipid content was quantified by relative fluorescence unit (RFU) of Nile red staining. Data were shown as mean ± SEM (*n* = 3/dose). ns, non-significant; * *p* < 0.05 statistical difference was calculated using one-way ANOVA followed by Tukey’s post hoc test.

OLE treatments were added at the concentration of 1, 10, 20, and 40 µM. Then, we used relative fluorescence unit (RFU) to quantify the lipid accumulation rate in hASCs (Figure 1A). Compared to undifferentiated cells, we observed significant lipid accumulation in all adipogenic-induced groups, both OLE-treated and nontreated. However, OLE-treated cells had significantly lower RFU compared to nontreated cells at concentrations of 20 µM and 40 µM. Induced hASCs treated with 20 and 40 µM OLE exhibited almost identical intensity values. Therefore, we decided on the OLE concentration of 20 µM for subsequent gene expression analysis.

### 2.3. Effects of OLE on Gene Expression Profiling during hASC Adipogenic Differentiation

**Figure 2:**
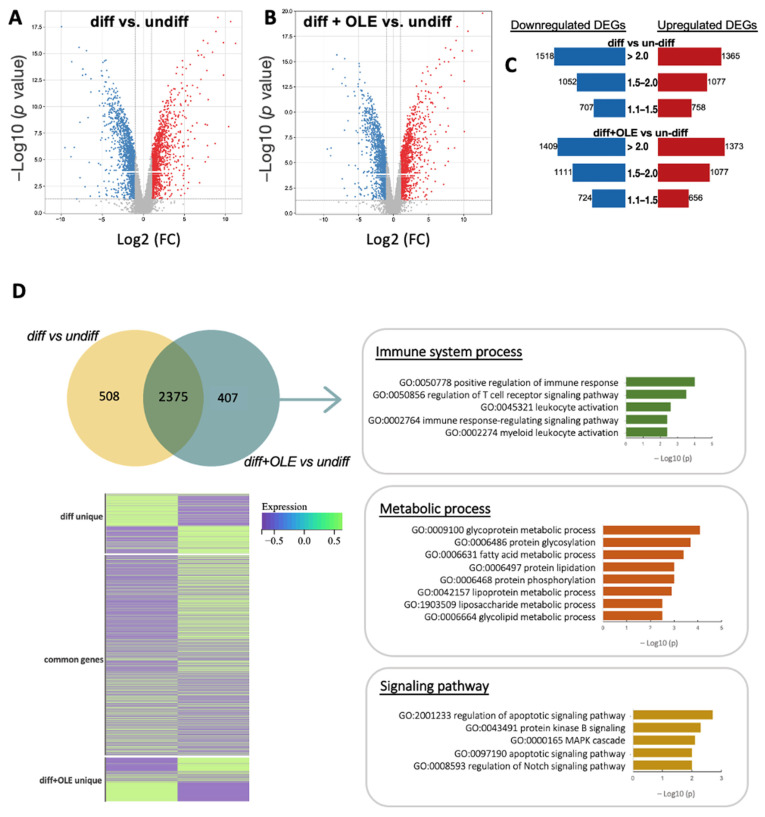
Characteristic of gene expression profiling in adipocyte with or without OLE. The volcano plots exhibit the DEGs, a comparison between differentiated adipocyte and undifferentiated adipose stem cells from hASCs (**A**), and a comparison between differentiated adipocytes with OLE and undifferentiated adipose stem cells from hASCs (**B**). The vertical axis (*y*-axis) corresponds to the *p*-value, and the horizontal axis (*x*-axis) displays log2 linear fold change. Up and downregulated DEGs are presented in the red and blue dots. (**C**) The bar graph displays the number of DEGs and the distribution of fold changes. The red bar represents the upregulated DEGs, and the blue bar represents downregulated DEGs. (**D**) Venn diagram showing the overlapped and unique DEGs in both conditions. GOBP results show the unique genes in differentiated with OLE group vs. undifferentiation group. The heatmap displays the DEGs intensity in two groups.

We performed whole-genome DNA microarray analysis to investigate the effects of OLE on gene expression profiling during hASC adipocytegenic differentiation. Genes with a 2-fold change (FC) and a significance level of *p* ≤ 0.05 were identified as differentially expressed genes (DEGs). The volcano plots display the FC values of all gene probe sets in the adipocyte-differentiated and undifferentiated group (diff vs. undiff), as well as the OLE-treated and adipocyte-differentiated and undifferentiated groups (diff + OLE vs. undiff) (Figure 2A,B). A total of 19,668 genes were identified in both comparison groups. In the hASCs adipocyte differentiation group, 2883 genes were differentially expressed compared to the undifferentiated group, of which 1365 DEGs were upregulated, and 1518 DEGs were downregulated. In the OLE-treated group, of 2782 DEGs, 1373 were upregulated, and 1409 were downregulated. Bar graphs show the distribution of FC values of up and downregulated DEGs (Figure 2C).

Moreover, we assessed the degree of overlap in the number of all DEGs in diff vs. un-diff and diff + OLE vs. undiff (Figure 2D). We identified 508 and 407 unique genes in diff vs. undiff and diff + OLE vs. undiff, respectively, and 2375 genes commonly regulated in both conditions. We checked the significantly enriched gene ontology biological processes (GOBP) by the common genes. We have identified some particular genes that were significant and display basic regulation, such as the regulation of proteolysis (GO:0030162), protein phosphorylation (GO:0006468), DNA metabolic process (GO:0006259), and the regulation of kinase activity (GO:0043549) in metabolic processes. Significantly enriched cellular processes included microtubule cytoskeleton organization (GO:0000226), cell population proliferation (GO:0008283), chromosome organization (GO:0051276), and mitotic cell cycle (GO:0000278) (Supplementary Figure S2).

Next, we checked the enriched GOBP terms with the 407 unique DEGs in diff + OLE vs. undiff to identify the OLE exclusive function. The parent terms, including immune system process (GO:0002376), metabolic process (GO:0008152), and signaling pathway (GO:0023052), were significantly enriched. For example, the positive regulation of immune response (GO:0050778) and the regulation of the T cell receptor signaling pathway (GO:0050856) were the significantly enriched immune system processes. The regulation of apoptotic signaling pathway (GO:2001233), protein kinase B signaling (GO:0043491), MAPK cascade (GO:0000165), and the regulation of Notch signaling pathway (GO:0008593) were the significantly enriched signaling pathways.

In addition, several enriched terms related to metabolic processes suggest that bioenergetics were more activated by the OLE treatment. These enriched terms were specifically associated with protein glycation processes, including glycoprotein metabolic processes (GO:0009100) and protein glycosylation (GO:0006486), as well as lipidation processes such as lipoprotein metabolic processes (GO:0042157) and lipid metabolism processes such as fatty acid metabolic processes (GO:0006631).

### 2.4. Gene Ontology Analysis of OLE-Treated hASCs Induced to Adipogenic Differentiation

**Figure 3:**
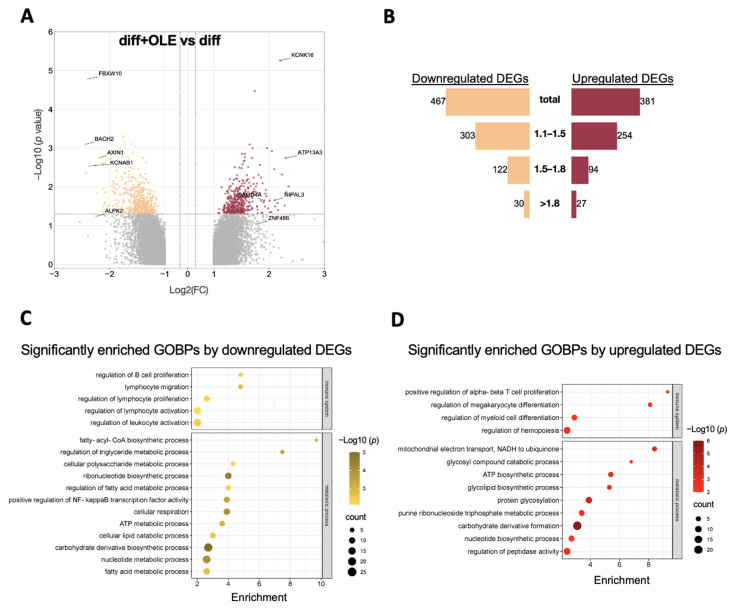
Microarray gene expression profile and GO analysis of OLE-treated adipocyte compared to nontreated adipocyte from hASCs. (**A**)The volcano plots exhibit the DEGs. The top 10 DEGs with the biggest fold changes are shown. The vertical axis (*y*-axis) corresponds to −log10 (*p*-value), and the horizontal axis (*x*-axis) displays log2 linear fold change. Up and downregulated DEGs are presented in the red and yellow dots. (**B**) The distribution of fold changes and the number of DEGs are shown in a bar graph. The blue bar represents DEGs that are downregulated, and the red bar represents DEGs that are upregulated. Dot plots are shown significantly enriched gene ontology biological process by downregulated DEGs (**C**) and upregulated DEGs (**D**). The *x*-axis displays the gene radio, and the *y*-axis represents the enrichment BPs, the size of bubbles pertains to the count/number of genes within the pathway, and the colors refer to the significance −log10 (*p*-value).

To clarify the molecular changes in adipocytes after adding OLE, we compared adipocytes with or without treatment (diff + OLE vs. diff). A total of 19,665 distinctive probe sets were recognized. The volcano plot is based on FC value with the criteria of 1.1-fold change (Figure 3A), and the bar graph illustrates the distribution of FC (Figure 3B). The top 10 up and downregulated DEGs were listed in detail (Table 1 and Table 2). A total of 848 genes were differentially expressed, with 381 upregulated DEGs and 467 downregulated DEGs. We identified the main GOBP terms with more than three qualifying genes. The GO terms for immune system processes (GO:0002376) and metabolic processes (GO:0008152) were examined.

Among these terms, the downregulated DEGs showed significant enrichment in terms related to the regulation of B cell proliferation and lymphocyte migration and activation. Additionally, the fatty-acyl-CoA biosynthetic process exhibited the highest enrichment score, followed by the triglyceride metabolic process and polysaccharide metabolic process. Moreover, terms related to the fatty acid metabolic process, cellular lipid catabolic process, and ATP metabolic process were all downregulated (Figure 3C). The carbohydrate biosynthetic process presented the most significant value, with the largest number of genes also enriched in the downregulated biological process (BP) results. In DEGs that were upregulated and enriched in BPs, several terms were observed, including T cell proliferation, megakaryocyte, and myeloid differentiation. Additionally, the biological processes of mitochondrial electron transport and glycolipid catabolic metabolism were identified. Notably, carbohydrate derivative formation again exhibited the highest significance and the largest number of genes, suggesting that OLE may impact carbohydrate metabolic processes.

### 2.5. OLE Exerted Anti-Inflammatory Effect and Regulated Lipid Metabolism in Adipocyte

**Figure 4:**
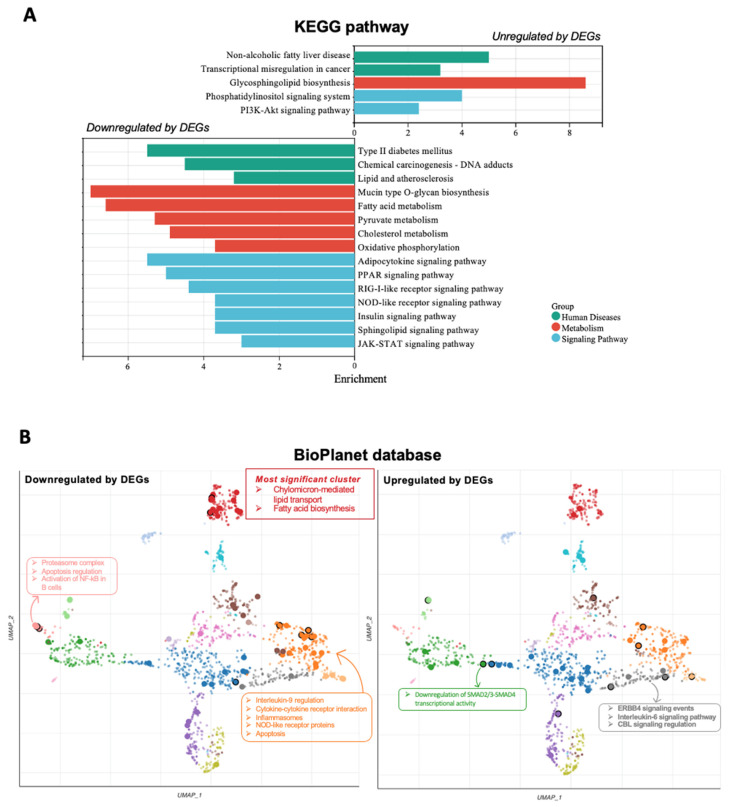
Pathway enrichment results. (**A**) Significantly enriched KEGG pathways in OLE-treated condition vs. non-treated condition. The vertical axis (*y*-axis) corresponds to terms, and the horizontal axis (*x*-axis) displays the enrichment score. (**B**) Scatterplot of all terms in the BioPlanet_2019 gene set library. Each term is represented by a single point. The points that are closer together indicate that the corresponding terms have more similar gene sets. The size and darkness of the points reflect the degree of enrichment significance, with larger and darker points indicating greater enrichment. For each gene set term, TF-IDF values were calculated and then reduced in dimensionality using the Uniform Manifold Approximation and Projection (UMAP) technique. The resulting terms were plotted using the first two UMAP dimensions on the scatter graph. The Leiden algorithm was applied to the TF-IDF values, leading to the identification of clusters. Each term was then assigned a color that corresponds to its respective cluster. Most significant terms in selected clusters are shown in the figure.

Next, we conducted pathway enrichment analysis to obtain a comprehensive and detailed pathway term list by using the Kyoto Encyclopedia of Genes and Genomes (KEGG) and BioPlanet_2019 database of diff + OLE vs. diff. The correlation among the enrichment score of various pathways in the KEGG database is shown in Figure 4A. It was observed that upregulated DEGs have a significant impact on nonalcoholic fatty liver disease and transcriptional misregulation in cancer pathways. The pathway of glycosphingolipid biosynthesis exhibited the highest enrichment score in the metabolism function. Furthermore, the upregulated DEGs showed enhancement of the phosphatidylinositol signaling system and P13K-Akt signaling pathway. The downregulated DEGs had a significant impact on various pathways, including T2D, which exhibited the highest enrichment score, followed by chemical carcinogenesis, lipid metabolism, and atherosclerosis in human diseases. In the metabolic function, the downregulated DEGs had a noticeable impact on pathways such as mucin-type O-glycan biosynthesis, fatty acid, pyruvate, cholesterol metabolism, and oxidative phosphorylation. Additionally, several signaling pathways, including adipocytokine signaling, PPAR signaling, insulin signaling, sphingolipid signaling, and the JAK-STAT signaling pathway, were regulated by the downregulated DEGs. The inhibition of certain pathways related to lipid metabolism by OLE indicates its potential beneficial effects in the treatment of type 2 diabetes (T2D).

**Figure 5:**
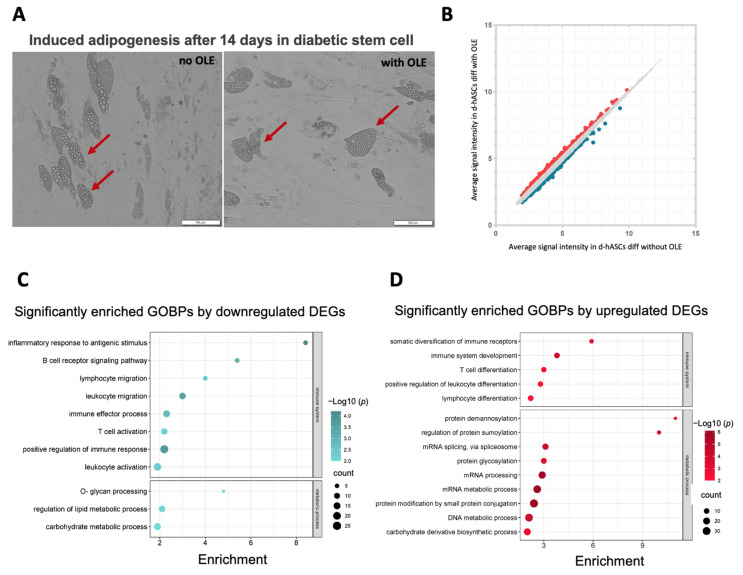
Microscopic images and gene expression analysis in adipocytes induced from d-hASCs after 14 days. (**A**) Representative light microscopic images of adipocyte after 14 days of induction with or without OLE. The arrows point to the lipid droplets in adipocytes. The scale bar indicates 100 µM. (**B**) Scatter plot showing the DEGs between OLE-treated and nontreated adipocytes from d-hASCs. The *x*-axis displays the average signal intensity of genes in adipocytes, and the *y*-axis corresponds to the average signal intensity of genes in OLE treated. Up and downregulated DEGs are presented in red and blue dots, respectively. Significantly enriched gene ontology biological process by downregulated DEGs (**C**) and upregulated DEGs (**D**). The gene ratio is shown on the *x*-axis, and the enrichment BPs are represented on the *y*-axis. The size of the bubbles corresponds to the number of genes in the pathway, and the colors indicate the significance of the −log10 (*p*-value).

To identify functionally clustered pathways, we used Uniform Manifold Approximation and Projection (UMAP) dimension reduction technique from the BioPlanet2019 database (Figure 5B). In the given analysis, the terms with similar gene sets are placed closer together. It was observed that the pattern of DEGs in both upregulated and downregulated clusters was the same but exhibited different significant terms. The downregulated DEGs exhibited the highest significant functions, which included chylomicron-mediated lipid transport and fatty acid biosynthesis represented by the color ‘red’. The downregulated DEGs showed highly enhanced pathways related to the proteasome complex, apoptosis regulation, and the activation of NF-kB in B cells, but these terms were not enriched in the upregulated DEGs. Additionally, the terms that were more enriched in the ‘orange’ cluster in the downregulated DEGs included interleukin-9 regulation, cytokine–cytokine receptor interaction, inflammasomes, NOD-LIKE receptor proteins, and apoptosis. However, in the upregulated DEGs clusters, the SMAD2/3-SMAD4 transcriptional activity showed significant enrichment, and the ‘gray’ points represent ERBB4 signaling events, interleukin-6 signaling, and CBL signaling regulation, which was also enriched in upregulated DEGs. The downregulation of DEGs resulting from the regulation of lipid and NF-κB transcription factor activity suggests that OLE not only regulates lipids but also exhibits anti-inflammatory effects on hASCs. Along with the upregulation of the CBL signaling pathway, the evidence indicates that OLE may also downregulate insulin resistance [27].

### 2.6. Effects of OLE on Gene Expression Profiling in Adipocytes Differentiated from Diabetic-hASCs

OLE treatment at 1 µM concentration for 14 days on d-hASCs adipocyte differentiation showed significantly smaller lipid droplets (Figure 5A). To further investigate the potential impact of OLE on adipogenesis in d-hASCs, we performed GO analysis to identify enriched BPs. Probes that exhibited an absolute value FC of 1.18 or higher and were sorted based on the top 500 DEGs in the OLE-treated group compared to the adipocyte differentiation group were selected. The distribution of all DEGs is illustrated in Figure 5B.

In terms of enriched BP identified through the GO analysis, the downregulated DEGs were notably enriched in immune system-related terms (Figure 5C). These included the inflammatory response to an antigenic stimulus, B cell receptor signaling pathway, lymphocyte migration, leukocyte activation and migration, immune effector process, and T cell activation, among others. Additionally, in terms of metabolic processes, the downregulated DEGs were involved in o-glycan processing, the regulation of lipid metabolic process, and carbohydrate metabolic process. The upregulated DEGs were found to be associated with several enriched BPs, including somatic diversification of immune receptors, immune system development, T cell differentiation, as well as leukocyte and lymphocyte differentiation. Moreover, in terms of metabolic processes, the upregulated DEGs were related to protein demannosylation, mRNA and DNA processing, and carbohydrate derivative biosynthetic processes.

The results of the study indicate that the regulation of lipid metabolism and anti-inflammatory effects observed in healthy adipocytes were also present in adipocytes obtained from hASCs. The use of OLE resulted in improved immune function-related terms, as evidenced by the downregulation of DEGs in adipocytes from d-hASCs. These findings suggest that OLE may have a potential regulatory role in these metabolic pathways.

### 2.7. Differential Effect of OLE on Adipocytes Differentiated from Healthy and Diabetic hASCs

**Figure 6:**
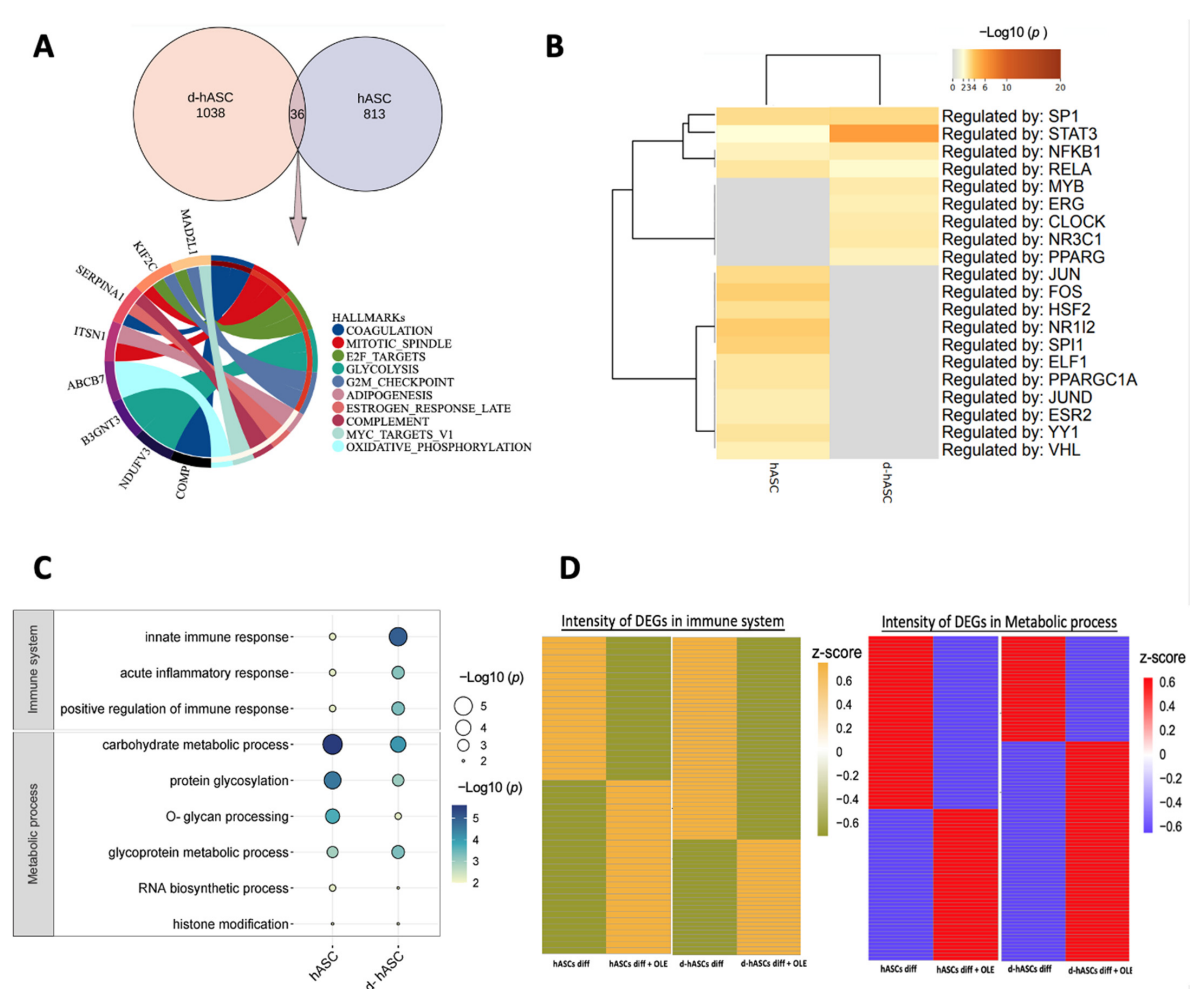
Comparison of DEGs in adipocytes differentiated from healthy and diabetic adipose stem cells. (**A**) Venn diagram showing the number of DEGs overlapped, and a circle plot displaying the hallmark gene sets enriched by the 36 overlapped DEGs. (**B**) Heatmap showing top 20 TF enrichment analysis in TRRUST. (**C**) A bubble plot displays the shared enrichment terms of OLE treatment in the two adipocytes. The color and the size of the circles indicate the −log10 (*p*-value). The terms are shown on the left. The group names are shown at the bottom. (**D**) Heatmap showing the intensity of the DEGs related to the immune system (**left**) and metabolic process (**right**). The color code represents the z-score (the integrated intensity value).

To find the target anti-inflammatory and metabolic functions of OLE, we performed a comparative analysis of adipocytes from healthy and diabetic conditions types. We overlapped the DEGs in adipocytes with OLE compared to nontreatment with OLE in hASCs and d-hASCs (Figure 6A). The adipocytes differentiated from d-hASCs exhibited 1038 unique genes, while the normal adipose cells showed 813 unique genes, with an overlap of 36 genes between the two conditions. These genes exhibited distinct and clearly defined biological states, displaying consistent expression patterns in adipogenesis, other types of differentiation (COAGULATION, MYC_TARGETS_V1), cell cycle regulation (E2F_TARGETS, G2M_CHECKPOINT, MITOTIC_SPINDLE), innate immune system function (COMPLEMENT), and glycolysis. Subsequently, we performed a transcription factor (TF) enrichment analysis using the TRRUST database (Figure 6B). In the top 20 TFs, half of the TFs differed, while four TFs, SP1(Specificity Protein 1), STAT3 (Signal Transducer and Activator of Transcription 3), NFκB1 (Nuclear Factor Kappa-Light-Chain-Enhancer of Activated B Cells 1) and RELA (v-Rel Avian Reticuloendotheliosis Viral Oncogene Homolog A) were same expressed with different significance in both groups. This implies that the four TFs may be the key to regulating genes related to health and diabetes adipocytes, which are related to anti-inflammation or lipogenesis.

Shared BPs, three from the immune system and six from metabolic processes, were identified (Figure 6C). The results indicate that OLE has a higher significance value in adipocytes differentiated from d-hASCs in the immune system, particularly in the innate immune response, acute inflammatory response, and regulation of immune response when compared to the hASCs group. In terms of metabolic processes, the highest value associated with carbohydrate metabolic processes was observed in the hASC group, while a smaller value was observed in the d-hASC group. This trend was also observed in protein glycosylation, O-glycan processing, and RNA biosynthetic process. Glycoprotein metabolic process and histone modification showed similar values in both groups. The confirmation of these points was supported by the intensity value (Figure 6D). In the adipocyte-induced in d-hASCs, a larger number of genes associated with inflammation were downregulated in comparison with hASCs. This shows a stronger anti-inflammatory function of OLE under disease conditions. It is noteworthy that the terms associated with metabolic processes in adipocytes from hASCs were more prominent than those in the d-hASCs group, but there were more upregulated genes in d-hASCs than in hASCs. This indicates that OLE is more effective in upregulating the metabolic processes in adipocytes from d-hASCs, particularly those related to carbohydrate and glycoprotein metabolism.

### 2.8. PPI Network Analysis of Adipocytes Differentiated from hASCs and d-hASCs

**Figure 7:**
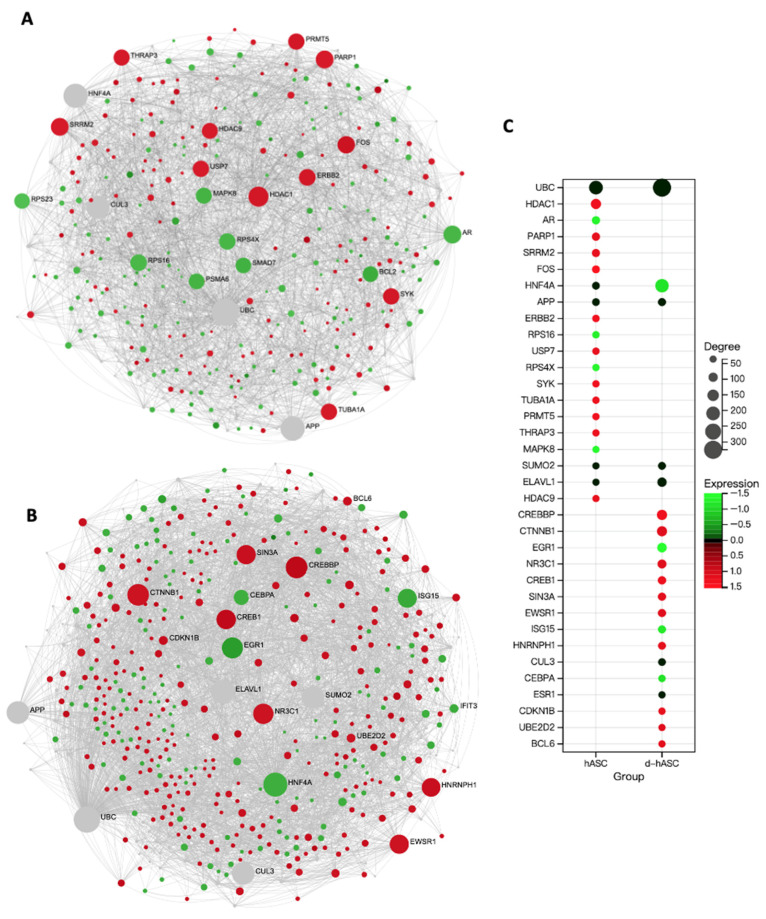
PPI Network analysis and molecular target prediction. Generic first-order PPI network of all DEGs in (**A**) hASCs and (**B**) d-hASCs. Conditions are shown in the ‘Force Atlas’ layout after the filtering. Each node represents the seeds and proteins, and edges between two connecting seeds indicate known interactions (IMEx Interactome database). Red nodes represent upregulated DEGs, and green nodes represent downregulated DEGs. Gray nodes are the proteins. (**C**) Top 20 seeds in the two adipocytes. The *y*-axis represents the name of each seed, the size of bubbles pertains to the degree value, and the colors refer to the expression value. The expression value for DEGs is the FC, and 0 for the predicted protein.

Next, the protein–protein interaction (PPI) network was curated to elucidate the links between expressed genes to find the connections in DEGs with potential proteins. The PPI analysis revealed 295 seeds with 1646 interacting nodes in adipocyte-hASC (Figure 7A) and 429 seeds with 923 nodes in adipocytes in d-hASCs groups (Figure 7B). The detailed top 20 seeds in each adipocyte with degree and expression value were listed (Figure 7C). There were five seeds shown in two adipocyte groups. The protein: ubiquitin C (UBC) was the top node with the highest degree in the two groups (degree = 208 in hASC, degree = 325 in d-hASC). We also provided the top 10 seeds list that appeared in the two adipocyte networks (Table 3 and Table 4), along with detailed information about each protein, including an enriched functional network. In both groups, UBC appears to be closely associated with regulating the activity of the NF-κB TF, protein phosphorylation, and the MAPK cascade. Additionally, hepatocyte nuclear factor 4 alpha (HFH4A), which is associated with lipid homeostasis and response to carbohydrate stimulus, was upregulated in the d-hASCs group. On the other hand, Amyloid Beta Precursor Protein (APP) was found to be linked to immune response. The majority of the hub genes identified were involved in RNA and DNA processing. This suggests that these genes may have a common role in the regulation of adipocyte function and metabolism, regardless of the presence of diabetes after OLE treatment. Additionally, this indicates that these genes could have important implications in the treatment of adipogenesis.

## 3. Discussion

In the present study, we have performed an integrated transcriptome analysis of OLE-treated adipocytes differentiated from hASCs and d-hASCs. We explored the prospects of OLE in anti-inflammatory functions and its effect on glucose and lipid metabolic functions.

The abnormal expansion and accumulation of adipocytes, resulting from an excessive differentiation of adipose stem cells towards an adipogenic lineage, have been linked to various health conditions such as obesity, insulin resistance, inflammation, metabolic disorders, and cardiovascular diseases [28,29]. Searching for compounds that are more efficient and effective in preventing adipocyte differentiation and promoting new adipocyte differentiation without depleting the reserve of stem cells has been an ongoing endeavor [30]. In this study, we explored adipocytes differentiated from both healthy and diabetic adipose-derived stem cells to investigate the impact of OLE on metabolic processes.

In hASCs, OLE exhibited normal proliferative ability in adipocytes, while the trend of lipid accumulation capacity decreased in adipocytes. Upon analyzing global microarray data under undifferentiated healthy adipose stem cell conditions, the unique genes showed significant enrichment in terms related to protein modification, such as protein phosphorylation, glycosylation, and lipidation. These modifications suggest that post-translational modifications occur in adipocytes upon treatment with OLE and regulate important cellular processes such as glucose uptake, lipid metabolism, adipocyte differentiation, insulin signaling, adipogenesis, lipolysis, and adipokine secretion [31,32]. We also found that the OLE downregulated fatty-acyl-CoA biosynthetic, triglyceride metabolic, and cellular lipid catabolic processes in adipocytes in BPs. Moreover, the findings from KEGG analysis indicated a downregulation of fatty acid metabolism and PPAR signaling pathway; meanwhile, the BioPlanet database highlighted the most significant cluster related to lipid transport. These results were supported by the research conducted by the Lepore team, who reported that OLE exhibited a protective effect against high-fat diet-induced adiposity in mice by reducing the levels of peroxisome proliferator-activated receptor gamma (PPARγ) and fatty acid synthase proteins [24]. All these results suggested that PPAR signaling may be a particular interest pathway, as the related genes directly impact lipid homeostasis.

Interestingly, T2D was the most highly enriched pathway in human diseases by downregulated DEGs, whereas nonalcoholic fatty liver disease was also prominently enriched, as indicated by the upregulated DEGs. We also observed a decrease in the expression of the adipokine signaling pathway in adipocytes differentiated from hASCs, which could indicate the presence of insulin resistance [33]. This case was also supported by an upregulation of the P13K-AKT signaling pathway in the KEGG result. Previous research has shown that the P13K-AKT pathway can downregulate insulin resistance, leading to improved glucose uptake, control of gluconeogenesis, and enhanced glycogen storage in a db/db mice model [34]. Despite this, even in the presence of insulin resistance, OLE treatment has the potential to preserve normal glucose metabolism in adipocytes through alternative pathways or transcription factors.

We also investigated the effects of OLE on adipocytes differentiated from d-hASCs. Treatment with a low concentration of OLE led to a reduction in adipocyte size and downregulation of lipid metabolic processes. These findings suggested that OLE has the potential to modify cellular processes related to adipocyte differentiation, which could have implications for diabetes treatment. This modulation of lipid metabolism is further supported by prior research carried out by our team employing adipocytes from d-hASCs on compounds with squalene derivatives [14]. Apart from these, more terms related to immune response were shown in the d-hASCs group. This is because, after adipocyte differentiation, adipose tissue inflammation attracts immune cells, which triggers the activation of resident macrophages and exacerbates insulin resistance and systemic inflammation [35]. Adipocytes are often inflamed due to pro-inflammatory cytokines in obese individuals, which can impact adipocyte function [36]. T cell proliferation was found to be positively regulated, while the migration and proliferation of other immune cells, such as B cells, lymphocytes, and leukocytes, were found to be downregulated, suggesting the presence of an immunosuppressive environment that could help prevent excessive inflammation in adipose tissue via OLE administration.

This phenomenon was also shown in the healthy adipocyte group. Not only was there a downregulation of T cells, B cells, lymphocytes, and leukocytes, but also the “NF-B transcription factor activity” term was downregulated in adipocytes differentiated from hASCs. Moreover, the DEGs regulated by NFκB1 and RELA were shown in two adipocytes that belong to the NF-κB family and play an important role in inflammation and metabolic disease [37]. Adipose tissue is known to be associated with chronic low-grade inflammation [38]. Previous research on human cells demonstrated that OLE amplifies anti-inflammatory activity in human macrophages via the expression of the CD163 receptor [39]. Additionally, in adipocytes with Simpson–Golabi–Behmel syndrome, OLE mitigated the expression of inflammation-related genes, resulting in decreased activation of NF-κB, which is consistent with our study [22,40]. OLE’s ability to rapidly activate signaling molecules through pharmacological principles may regulate the nutritional modulation of inflammatory diseases [41]. All these findings revealed that certain inflammation-related functions could be regulated in adipocytes by OLE.

For finding the potential target of the OLE effect in adipocytes from healthy and diabetic stem cells, DEGs related to TFs and their’ PPI networks were checked. Various genes involved in the transcriptional regulation of adipogenesis differentiation were induced by OLE treatment. Our TF analysis revealed that SP1 was the most abundant regulator of DEGs, displaying the highest significance in both groups. SP1 is known to activate the transcription of the glucose transporter Glut-1, which plays a crucial role in glucose uptake in various cell types, including adipocytes [42]. Additionally, emerging evidence suggests that both SP1 and STAT3 can suppress the expression of pivotal genes implicated in adipocyte differentiation, including PPARγ, C/EBPα, and FABP4, thereby potentially restoring insulin sensitivity and mitigating obesity [43,44]. Additionally, SUMOylation (SUMO) was identified as a regulator of PPARγ activity in our PPI analysis, offering a potential strategy for developing safer drugs for treating type 2 diabetes by inhibiting insulin-sensitizing activity without affecting adiposity in mice [45]. ELAVL1 binds to a cyclic nucleotide-dependent stabilizing domain in the 3′ untranslated region of Na(+)/glucose cotransporter mRNA. The drugs inferred for the *ELAVL1* gene are reported to affect glucocorticoids, anti-inflammatory agents, small molecules, and anti-inflammatory glucocorticoids [46]. Based on these findings, it is possible to hypothesize that although the upregulation of the P13K-AKT pathway in hASCs may lead to insulin resistance, the identified targets have the potential to restore normal glucose metabolism through alternative pathways. This suggests that these targets could help mitigate insulin resistance.

Furthermore, while the effects of OLE on lipid and glucose metabolism, as well as immune response, remained consistent, our results revealed variations between the two sources of differentiated adipocytes. STAT3 participates in various cellular processes and is involved in the regulation of carbohydrate and lipid metabolism [47]. Despite the fact that STAT3 is regulated in both groups, its expression level is higher in the d-hASC group due to the activation of STAT3 by pro-inflammatory cytokines, increasing its levels, as has been observed in significantly upregulated T2D models [48]. Additionally, we found that PPARγ was only shown to control DEGs in the d-hASCs group, which are concerned with T2D risk through the involvement in adipocyte differentiation and energy homeostasis in a previous study [49]. Moreover, the exclusive presence of *HNF4A* expression in adipocytes derived from d-hASCs, but not in the hASCs group, highlights its strong association with the “lipid metabolism” in one human research related to diabetes [50]. This gene also holds potential as a candidate gene for type 2 diabetes in individuals with severe obesity [51]. The unique expression patterns observed suggest that these genes may serve as potential targets for regulating lipid metabolism in adipocytes derived from diabetic adipose stem cells, requiring further investigation.

According to structure characteristics among secoiridoid derivatives in EVOO, OLE, derived from oleuropein, possesses a secoiridoid aglycone structure similar to that of oleuropein [52]. Previous studies reported that oleuropein aglycone exhibited diverse pharmacological effects, including anti-hyperglycemic and lipid-lowering properties [53], as well as the ability to enhance UCP1 expression in brown adipose tissue, thereby activating β-adrenergic signaling in obese rats induced by a high-fat diet [54]. In our study, we identified alterations in terms that belong to lipid and glucose metabolism, as well as ATP biosynthetic processes, in adipocytes derived from healthy adipose stem cells. These findings suggest a potential involvement of the aglycone structure of OLE in the regulation metabolism on fat accumulation and glucose. Additionally, it is noteworthy that oleuropein aglycone exhibited notable stability under acidic conditions simulating gastric digestion, with limited hydrolysis observed [55,56]. Similarly, OLE demonstrated favorable stability in a gastric digestion model [56,57]. Here, we utilized OLE by employing a one-step synthesis method from oleuropein, which demonstrated higher efficiency compared with the more complex over 10-step synthesis method [58] and the use of H-mont did not result in any decrease in the yield of OLE [20]. This suggests that the stable and highly pure structure of OLE may have a significant role in regulating adipocyte fat and glucose metabolism.

It is important to note that our findings are derived from whole-genome transcriptome-level microarray data, offering preliminary insights for future investigations, particularly concerning adipocytes derived from diverse sources. Most findings are consistent with previous studies regarding adipocyte metabolic homeostasis and immune response. Further in vitro studies are necessary to investigate the potential targets and preventive effects of OLE, as well as to elucidate the structural characteristics of OLE in relation to its functional properties. Meanwhile, in vivo studies focusing on diabetic model treatment are required to confirm the potential functionalities of OLE observed in the present study.

**Figure 8:**
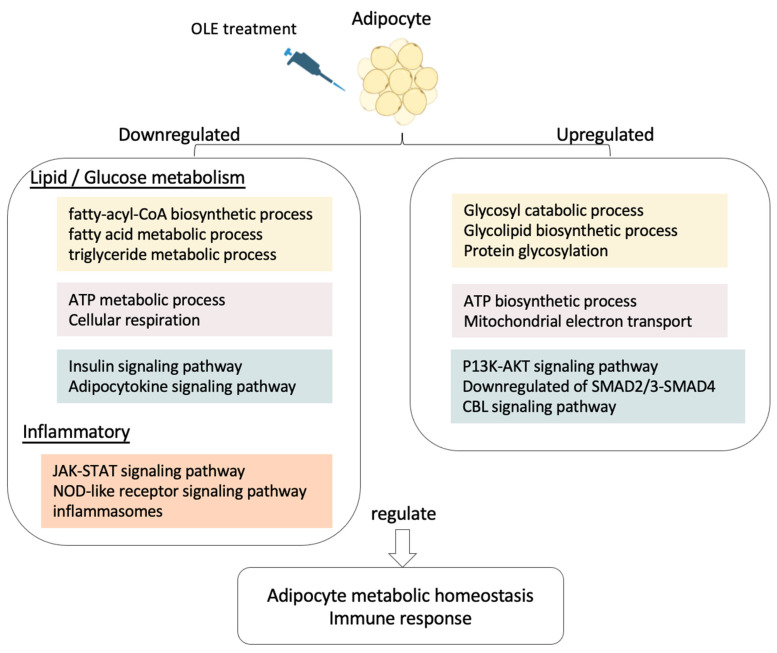
Summary of biological events regulated by OLE in adipocytes differentiated from hASCs.

Based on the latest research, the link to metabolic processes in OLE, such as glucose and lipid metabolism, as well as chroma-tin-modifying enzymatic activities, through computational de-orphanization (a structure-based tool) has been experimentally validated [59]. In our study, it appears that OLE promotes adipocyte metabolism in both hASCs and d-hASCs. Additionally, OLE has been shown to regulate lipid and glucose metabolic functions and enhance anti-inflammatory properties during adipocyte differentiation. This is achieved by balancing glucose metabolism through glycosides and glycolipids (Figure 8). OLE has the potential to aid in the remedy of insulin resistance and prevent the onset of early T2D. Overall, our findings provide a promising foundation for the development of a novel class of therapeutics derived from natural compounds.

## 4. Materials and Methods

### 4.1. Chemicals

OLE was chemically synthesized from oleuropein using proton-exchanged montmorillonite as a catalyst and used after column separation and freeze-drying [20]. Briefly, oleuropein powder (1.53 g, purity = 88.0%) was treated in the presence of proton-exchanged montmorillonite (3.06 g) in DMSO (10.0 mL) containing 7.16 mmol of H_2_O at 150 °C for 3 h without stirring. After removing the catalyst by filtration, the crude products were extracted with AcOEt/H_2_O. OLE was isolated via column chromatography at a yield of 75% (0.598 g).

### 4.2. Cells Culture and Techniques

hASCs were purchased from ATCC^®^ (SCRC-4000TM, Manassas, VA, USA). These cells were isolated from the adipose tissue of a Caucasian female. Cell culture was performed using a modified process based on the previous description [60,61]. Briefly, the cells were cultured in a DMEM/F12 (11320033, Thermo Fisher Scientific, Waltham, MA, USA) based medium. This medium consisted of 10% (*v*/*v*) fetal bovine serum (FBS), 1% (*v*/*v*) penicillin/streptomycin, 1% GlutaMax (35050061, Thermo Fisher Scientific), and 1‰ (*v*/*v*) 2-mercaptoethanol (21985023, Thermo Fisher Scientific, USA), under conditions at 37 °C in a 5% CO_2_ environment. The cells were seeded at a density of 5000 viable cells per cm^2^ and passaged after reaching 80% confluence as recommended by the provider.

d-hASCs were purchased from Lonza (PT-5008, Walkersville, MD, USA). Detailed culture conditions have been explained in our previous study [14]. Briefly, the cells were cultured at 37 °C in a 5% CO_2_ humidified incubator using ASC growth medium sourced from the Lonza company. The ASC growth medium comprised ASC basal medium supplemented with 10% FBS, 1% L-glutamine, and gentamicin–amphotericin B (GA-1000), following the manufacturer’s instructions.

### 4.3. Adipocyte Differentiation of hASCs and d-hASCs

White adipocyte differentiation of hASCs was performed using the DMEM/F12-based medium as previously described according to the regularly used protocol with slight modifications to ensure successful adipocyte induction [62]. Adipogenic differentiation was initiated on the second day after cell confluence, followed by a 7-day induction period and a 14-day maintenance period. The induction cocktail contained 100 nM insulin (093-06351, Wako, Japan), 1 μM dexamethasone (10008980, Cayman Chemical, Ann Arbor, MI, USA), 0.5 mM IBMX (10008978, Cayman Chemical, USA), and 1 μM rosiglitazone. For the maintenance medium, 100 nM insulin and 1 μM dexamethasone were prepared with a DMEM-F12-based medium. To generate white adipocytes, hASCs were seeded at a density of 5 × 10^4^ cells per cm^2^ and grown to confluence. Both the induction and maintenance medium was subsequently changed every 4 days for a total of 21 days.

For induced adipocytes from d-hASCs, the cells were seeded in the preadipocyte growth medium (PGM-2 ^TM^ Bulletkit, Lonza, Walkersville, MD, USA). When the cells reach 80% confluency, change the growth medium to adipogenic differentiation medium to induce adipogenicity. Rhinsulin, dexamethasone, IBMX, and indomethacin were added to the adipogenic differentiation medium. Refresh the medium every 10 to 12 days [14].

### 4.4. Cell Viability Assay

The cell viability of the hASCs was analyzed using the 3-(4,5-Dimethylthiazol-2-yl)-2,5-diphenyltetrazolium bromide (MTT) assay. Succinate dehydrogenase in the mitochondria of living cells can reduce exogenous MTT to water-insoluble formazan and deposit it in cells, whereas dead cells have no such function. In brief, cells at the density of 5 × 10^4^ cells/mL were seeded in a 96-well plate. After 16 h, the cells were treated with oleacein concentrations of 0, 1, 5, 10, 20, 40, 80, and 100 μM for 24 h and then incubated with MTT (0.5 mg/mL) for an additional 4 h. The forming formazan was dissolved using 10% SDS. Using a microplate reader, the absorbance was read at 570 nm. We conducted three independent experiments. Data were calculated as cell proliferation (%) = (mean OD value of treated well − mean OD value of the blank)/(mean OD value of untreated well − mean OD value of the blank) × 100%.

### 4.5. Cell Measurement and Lipid Accumulation Assay on hASCs

The hASC cells were subjected to an induction medium to induce adipocyte differentiation, which was followed by a maintenance medium in both 37 °C and 5% concentrations of CO_2_ environments. Lipid droplet formation was observed using the Leica TCS SP8 confocal microscope, and the cellular lipid accumulation was determined using Nile red (144-08811, FUJIFILM, Tokyo, Japan). The Nile red working solution concentration was 25 μg/mL by dissolving in DMSO. To quantify lipid accumulation, cells were seeded in a black 96-well plate and differentiated into adipocytes for 21 days. After washing with PBS, 5 μL of the working solution was added to each well containing PBS and incubated for 30 min at 37 °C. Fluorescence was measured by using a microplate reader (Varioskan LUX, Thermo Fisher Scientific, Waltham, MA, USA) with excitation at 485 nm and emission at 535 nm, according to the manufacturer’s introduction. For each concentration, we conducted three independent experiments. RFU was used to quantify the fluorescence intensity emitted by the Nile Red. The data were calculated as RFU = [(mean RFU value of treated well − mean RFU value of blank) × 100%]/(mean RFU value of untreated well − mean RFU value of blank).

### 4.6. RNA Extraction and Quantification

The cells were cultured in a 6-well plate with total volumes of 3 mL during induction and maintenance medium conditions. An Isogen kit (Nippon Gene, Tokyo, Japan) was used to extract the total RNA according to the manufacturer’s instructions. RNA was quantified using the Nanodrop 2000 spectrophotometer (ThermoScientific, Waltham, MA, USA).

### 4.7. Microarray Experiment Processing

The GeneChip 3′IVT PLUS Reagent Kit (Affymetrix Inc., Santa Clara, CA, USA) and Affymetrix^®^ 3′ IVT Array Strips for Affymetrix’s GeneAtlas^®^ System were utilized for the gene expression profiling of d-hASC, which was carried out following the instructions in the manufacturer’s user guide. As previously described, the total RNA was extracted, and then 250 ng per sample was used for strand synthesis. GeneAtlas^®^ Hybridization station, GeneAtlas^®^ Fluidics Station, and GeneAtlas^®^ Imaging Station were used for hybridization, washing, staining, and scanning for the human genome array strips after the samples were fragmented and labeled. For the hASC group, we used Clariom S Assay Human with the SST-RMA algorithm, and for the d-hASC group, we used Human GENO-U219 with the RMA algorithm.

### 4.8. Gene Expression Analysis

The probe intensity for various comparisons was conducted using Transcriptome Analysis Console software (version 4.0.2, Thermo Fisher Scientific, Waltham, MA, USA) A threshold value of FC > 2 (in linear space) and a *p*-value of < 0.05 (one-way between-subject analysis of variance ANOVA) were set to identify differentially expressed genes (DEGs) in analyzing the comparisons to normal hASCs (undiff). After adipocyte differentiation, DEGs were filtered using a linear fold change (FC) greater than 1.1 and a *p*-value less than 0.05 for comparing the OLE + diff and diff groups in hASCs. For DEGs in d-hASCs, the FC values of the top 500 genes (FC = 1.18) were extracted and sorted in descending order for clustering and more focused enrichment analysis.

Metascape (http://metascape.org, accessed on 1 March 2023), a web-based tool, was utilized to conduct gene ontology (GO) and Kyoto Encyclopedia of Genes and Genomes (KEGG) pathway enrichment analyses [63]. The BioPlanet_2019 database was used to cluster genes function under the Enrichr online tool (https://maayanlab.cloud/Enrichr/, accessed on 9 March 2023) [64,65,66]. The transcription factor (TF) and DEG connections were examined using TRRUST (transcriptional regulatory relationships unraveled by sentence-based text-mining)) [67] and PPI networks, using the PPI networks constructed via the NetworkAnalyst tool (https://www.networkanalyst.ca/NetworkAnalyst/home.xhtml, accessed on 27 March 2023) [68], which utilizes the IMEx Interactome database [69]. The initial set of seeds and nodes was constructed based on the first-order generic PPI. We set the degree filter value as 3.0 and the tissue filter as adipose tissue and manually minimized the network more specifically. A bioinformatics online tool (https://www.bioinformatics.com.cn/, accessed on 15 March 2023) was used to generate the balloon plot and bubble plot, while the lollipop illustration was visualized using Sangerbox 3.0 together with the circle plot under the condition of FDR based on Benjamini–Hochberg (http://sangerbox.com/, accessed on 27 March 2023) [70].

### 4.9. Statistical Analysis

One-way ANOVA was performed using GraphPad Prism 9 software (San Diego, CA, USA) to determine statistical significance. A *p*-value of 0.05 was considered significant. The results are reported as mean ± SEM. The results represent the average of three parallel experiments.

## 5. Conclusions

In summary, our findings suggest that OLE has the potential to confer health benefits, particularly with regard to reducing inflammation and maintaining metabolic homeostasis in adipocytes differentiated from both healthy and diabetic adipose stem cells. Moreover, we have identified potential targets of OLE that regulate lipid and glucose metabolism. While our study is under the whole-genome transcriptomics analysis, it provides a basis for future investigations into the therapeutic potential of OLE in the fields of chemical biology and drug discovery.

## Figures and Tables

**Table 1:** List of the top significantly upregulated genes and biological functions.

Gene Symbol	Description	Fold Change	*p* Value	Function
*ATP13A3*	ATPase 13A3	2.7	0.0089	ATP Binding, Intracellular Calcium Ion Homeostasis, Monoatomic Cation Transmembrane Transport, Polyamine Transmembrane Transport
*KCNK16*	Potassium Two Pore Domain Channel Subfamily K Member 16	2.28	4.91 × 10^−5^	Potassium Channel Activity, Protein Binding, Stabilization Of Membrane Potential, Regulation Of Monoatomic Ion Transmembrane Transport, Plasma Membrane
*ZNF486*	Zinc Finger Protein 486	2.08	0.0299	DNA-Binding Transcription Factor Activity, RNA Polymerase II-Specific, Metal Ion Binding, Regulation Of DNA-Templated Transcription
*SAMD4A*	Sterile Alpha Motif Domain Containing 4A	2.08	0.0207	RNA Binding, mRNA Binding, Protein Binding, Translation Repressor Activity
*NIPAL3*	NIPA-Like Domain Contains 3	2.07	0.0089	Protein Binding, Magnesium Ion Transmembrane Transporter Activity, Magnesium Ion Transport, Magnesium Ion Transmembrane Transport
*GNG7*	G Protein Subunit Gamma 7	2.07	0.0203	Protein Binding, Receptor Guanylyl Cyclase Signaling Pathway, G Protein-Coupled Receptor Signaling Pathway, Regulation Of Adenylate Cyclase Activity
*SWI5*	SWI5 Homologous Recombination Repair Protein	2.06	0.0017	Protein Binding, Double-Strand Break Repair Via Homologous Recombination, Cellular Response To Ionizing Radiation
*LY6K*	Lymphocyte Antigen 6 Family Member K	2.05	0.0269	Acrosomal Vesicle, Extracellular Region, Plasma Membrane, Membrane Raft
*INPP5F*	Inositol Polyphosphate-5-Phosphatase F	2.04	0.0236	Protein Binding, Inositol Monophosphate 1-Phosphatase Activity, Protein Homodimerization Activity, Positive Regulation Of Receptor Recycling, Phosphatidylinositol Biosynthetic Process, Phosphatidylinositol Catabolic Process, Phosphatidylinositol Dephosphorylation
*ERBB4*	Erb-B2 Receptor Tyrosine Kinase 4	2.04	0.0044	Protein Binding, ATP Binding, Signal Transduction, Transmembrane Receptor Protein Tyrosine Kinase Signaling Pathway, Nervous System Development, ERBB4 Signaling Pathway, Mitochondrial Fragmentation Involved In Apoptotic Process, Positive Regulation Of Receptor Signaling Pathway Via JAK-STAT, Protein Autophosphorylation

**Table 2:** List of the top significantly downregulated genes and biological functions.

Gene Symbol	Description	Fold Change	*p* Value	Function
*BACH2*	BTB Domain and CNC Homolog 2	−2.83	0.002	RNA Polymerase II Cis-Regulatory Region Sequence-Specific DNA Binding, Protein Binding, Sequence-Specific Double-Stranded DNA Binding, Primary Adaptive Immune Response Involving T Cells, Additionally, B Cells
*KCNAB1*	Potassium Voltage-Gated Channel Subfamily A Regulatory Beta Subunit 1	−2.38	0.0074	Voltage-Gated Potassium Channel Activity, Potassium Channel Regulator Activity, Protein Domain Specific Binding, Transmembrane Transporter Binding, NADPH Binding, Molecular Function Inhibitor Activity, Potassium Ion Transport
*AXIN1*	Axin 1	−2.31	0.0003	P53 Binding, Protein Binding, Enzyme Binding, Protein Polyubiquitination, Positive Regulation of Transforming Growth Factor Beta Receptor Signaling Pathway, Regulation of Protein Ubiquitination, Regulation of Fat Cell Differentiation, Canonical Wnt Signaling Pathway
*ALPK2*	Alpha Kinase 2	−2.28	0.0225	Protein Serine/Threonine Kinase Activity, ATP Binding, Protein Phosphorylation, Regulation of Gene Expression, Establishment of Cell Polarity, Regulation of Apoptotic Process
*FBXW10*	F-Box and WD Repeat Domain Containing 10	−2.27	5.01 × 10^−5^	Cytosol
*MYEF2*	Myelin Expression Factor 2	−2.16	0.0388	DNA Binding, RNA Binding, mRNA Binding, Myotube Differentiation, Neuron Differentiation, Nucleus
*ITSN1*	InterSection 1	−2.12	0.0411	Guanyl-Nucleotide Exchange Factor Activity, Calcium Ion Binding, Protein Binding, Molecular Adaptor Activity, Molecular Adaptor Activity, Exocytosis, Protein Localization, Protein Transport, Endosomal Transport, Intracellular Signal Transduction, Regulation of Small Gtpase Mediated Signal Transduction, Clathrin-Dependent Synaptic Vesicle Endocytosis
*CHAC2*	ChaC Glutathione Specific Gamma Glutamylcyclotransferase 2	−2.09	0.0153	Gamma-Glutamylcyclotransferase Activity, Glutathione Biosynthetic Process, Glutathione Catabolic Process
*L3MBTL4*	L3MBTL Histone Methyl-Lysine Binding Protein 4	−2.08	0.0471	Protein Binding, Zinc Ion Binding, Chromatin Organization
*IL7*	Interleukin 7	−2.07	0.0038	Cytokine Activity, Interleukin-7 Receptor Binding, Protein Binding, Growth Factor Activity, Cell–cell Signaling, Cytokine-Mediated Signaling Pathway, B Cell Proliferation

**Table 3:** List of the top 10 hub genes in OLE-treated adipocytes induced from hASCs and enriched functional network.

Seeds	Description	Degree	Expression	Enriched Functional Network
*HDAC1*	Histone Deacetylase 1	116	1.38	Transcription initiation from RNA polymerase II promoter, Histone modification, Negative regulation of the apoptotic process
*AR*	Androgen Receptor	67	−1.38	transcription from RNA polymerase III promoter, regulation of sequence-specific DNA binding transcription factor activity, regulation of MAPK cascade
*PARP1*	Poly(ADP-Ribose) Polymerase	64	1.25	Protein processing, transforming growth factor beta receptor signaling pathway, glycoprotein biosynthetic process, regulation of transcription from RNA polymerase II promoter
*SRRM2*	Serine/Arginine Repetitive Matrix 2	63	1.26	mRNA processing, nucleobase containing compound metabolic process, cellular aromatic compound metabolic process
*FOS*	Fos Proto-Oncogene, AP-1 Transcription Factor Subunit	57	1.54	Transforming growth factor beta receptor signaling pathway, response to steroid hormone stimulus, response to oxidative stress, inflammatory response, MAPK cascade
*ERBB2*	Erb-B2 Receptor Tyrosine Kinase 2	45	1.39	Positive regulation of translation, T cell proliferation, protein autophosphorylation, regulation of immune system process, lymphocyte differentiation, regulation of MAPK cascade
*RPS16*	Ribosomal Protein S16	44	−1.33	Cellular protein complex disassembly, RNA catabolic process, ribonucleoprotein complex biogenesis
*USP7*	Ubiquitin Specific Peptidase 7	41	1.27	DNA modification, Cellular protein catabolic process, DNA repair, Protein catabolic process
*RPS4X*	Ribosomal Protein S4 X-Linked	41	−1.39	Regulation of translation, Protein targeting to membrane, Cellular protein complex disassembly, RNA catabolic process
*SYK*	Spleen Associated Tyrosine Kinase	40	1.39	Activation of JUN kinase activity, Superoxide metabolic process, regulation of cytokine secretion, regulation of T cell proliferation, regulation of cell–cell adhesion, Activation of MAPK activity, Lipid transport, Fatty acid metabolic process, Activation of immune response

**Table 4:** List of the top 10 hub genes in OLE-treated adipocytes induced from d-hASCs and enriched functional network.

Seeds	Description	Degree	Expression	Enriched Functional Network
*HNF4A*	Hepatocyte Nuclear Factor 4 Alpha	202	−1.19	Lipid homeostasis, response to carbohydrate stimulus, regulation of lipid metabolic process, cellular amino acid metabolic process
*CREBBP*	CREB Binding Protein	119	1.38	Transcription initiation from RNA polymerase II promoter, histone modification, regulation of cell differentiation, innate immune response
*CTNNB1*	Catenin Beta 1	116	1.18	Proteoglycan metabolic process, regulation of T cell proliferation, lymphocyte activation, regulation of I-κB kinase/ NF-κB cascade, muscle cell differentiation, regulation of MAPK cascade, glycoprotein biosynthetic process
*EGR1*	Early Growth Response 1	101	−1.51	T cell differentiation, lymphocyte differentiation, cytokine-mediated signaling pathway, leukocyte differentiation
*NR3C1*	Nuclear Receptor Subfamily 3 Group C Member 1	84	1.18	Steroid biosynthetic process, carbohydrate biosynthetic process, transcription initiation from RNA polymerase II promoter, lipid metabolic process, glucose metabolic process
*CREB1*	CAMP Responsive Element Binding Protein 1	67	1.33	Phosphatidylinositol mediated signaling, regulation of lipid metabolic process, regulation of immune response, MAPK cascade
*SIN3A*	SIN3 Transcription Regulator Family Member A	63	1.22	Histone modification, immune effector process
*EWSR1*	EWS RNA Binding Protein 1	62	1.18	Regulation of transcription, regulation of RNA metabolic process
*ISG15*	ISG15 Ubiquitin-Like Modifier	59	−1.26	Response to virus, regulation of cytokine production, cellular protein catabolic process, immune effector process, protein catabolic process
*HNRNPH1*	Heterogeneous Nuclear Ribonucleoprotein H1	55	1.24	RNA splicing, mRNA processing, regulation of RNA metabolic process, regulation of gene expression, regulation of cellular metabolic process

## Data Availability

The supporting data of this article can be found within this paper and in supplementary files. The microarray data have been deposited in the NCBI GEO database and are publicly available (Accession number: GSE230618 (hASCs) and GSE230619 (d-hASCs); GSE230618 (https://www.ncbi.nlm.nih.gov/geo/query/acc.cgi?acc=GSE230618, accessed on 26 April 2023). GSE230619 (https://www.ncbi.nlm.nih.gov/geo/query/acc.cgi?acc=GSE230619, accessed on 26 April 2023).

## Supplementary Materials

The following supporting information can be downloaded at: https://www.mdpi.com/article/10.3390/ijms241310419/s1.
