# Supplementary figures and images for: A Rare Olive Compound Oleacein Improves Lipid and Glucose Metabolism, and Inflammatory Functions: A Comprehensive Whole-Genome Transcriptomics Analysis in Adipocytes Differentiated from Healthy and Diabetic Adipose Stem Cells
Source: Int J Mol Sci. 2023 Jun 21;24(13):10419. doi: 10.3390/ijms241310419 (PMC10341505; doi:10.3390/ijms241310419)

## Supplementary Figure S1

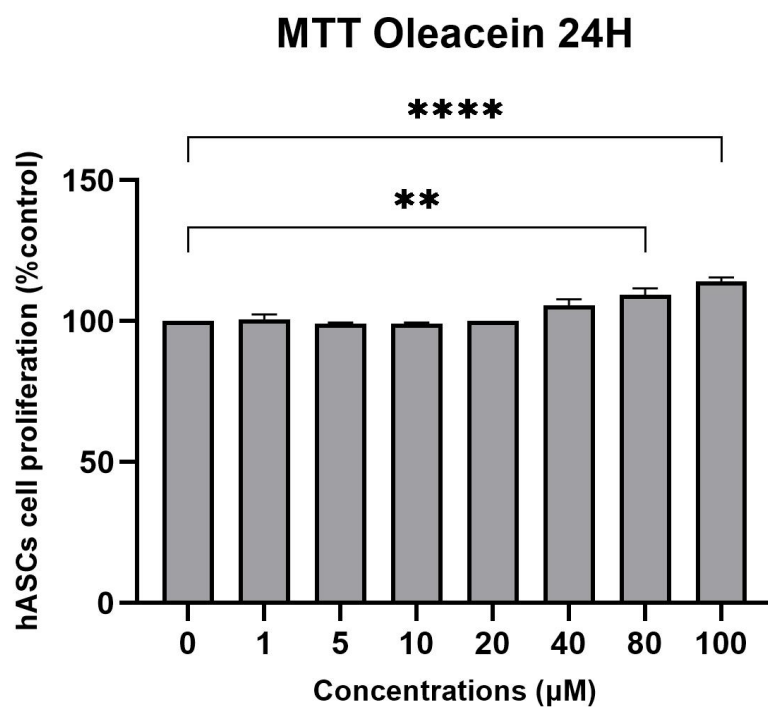

Supplementary Figure S2

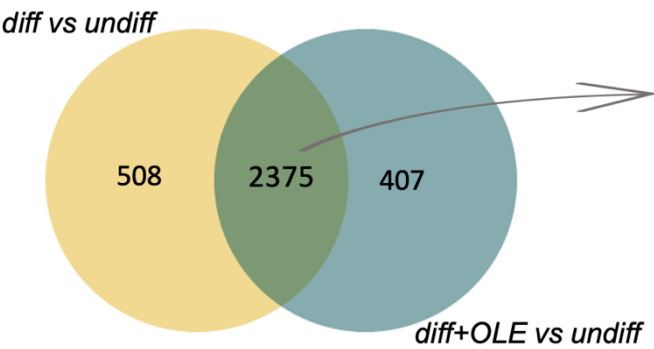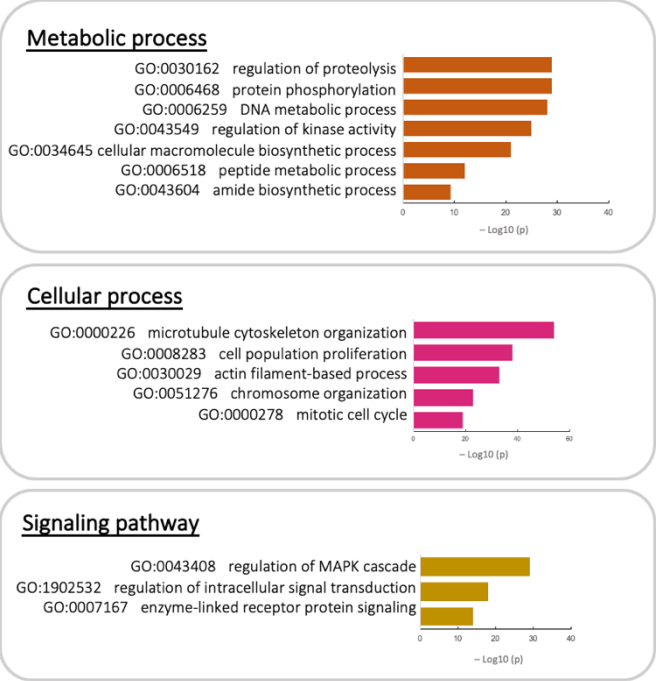

Supplement: Supplementary file 1 [file ijms-24-10419-s001.zip › ijms-2441136-supplementary.pdf]
